# Lessons from 13 years of accelerometry measurements in five Brazilian cohorts: methodological aspects

**DOI:** 10.1590/0102-311XEN011724

**Published:** 2025-04-11

**Authors:** Andrea Wendt, Giulia Salaberry Leite, Renata Contreira, Grégore Iven Mielke, Bernardo Lessa Horta, Janaína Vieira dos Santos Motta, Fernando Pires Hartwig, Fernando César Wehrmeister, Ana Maria Baptista Menezes, Helen Gonçalves, Iná S. Santos, Alicia Matijasevich, Luciana Tovo-Rodrigues, Andréa Dâmaso Bertoldi, Marlos Domingues, Renata Moraes Bielemann, Ulf Ekelund, Pedro C. Hallal, Inácio Crochemore-Silva

**Affiliations:** 1 Programa de Pós-graduação em Tecnologia em Saúde, Pontifícia Universidade Católica do Paraná, Curitiba, Brasil.; 2 Programa de Pós-graduação em Epidemiologia, Universidade Federal de Pelotas, Pelotas, Brasil.; 3 School of Public Health, The University of Queensland, Brisbane, Australia.; 4 Departamento de Medicina Social, Universidade Federal de Pelotas, Pelotas, Brasil.; 5 Faculdade de Medicina, Universidade de São Paulo, São Paulo, Brasil.; 6 Department of Sports Medicine, Norwegian School of Sport Sciences, Oslo, Norway.; 7 Department of Chronic Diseases, Norwegian Institute of Public Health, Oslo, Norway.; 8 College of Applied Health Sciences, University of Illinois Urbana-Champaign, Champaign, U.S.A.

**Keywords:** Accelerometry, Cohorts Studies, Methods, Developing Countries, Acelerometria, Estudos de Coortes, Métodos, Países em Desenvolvimento, Acelerometría, Estudios de Cohortes, Métodos, Países en Desarrollo

## Abstract

This study describes key methodological decisions and their justifications for accelerometer data collection, processing, and cleaning/analysis in Pelotas (Rio Grande do Sul State, Brazil) cohorts, exemplifying how research using sensor monitors could be carried out in a middle-income country context. This is a descriptive methodological study using raw accelerometer data from five Brazilian population-based cohorts with 32,963 individuals. Data collection (pre-processing decisions), processing (choosing requirements to run the analysis), and post-processing decisions (data cleaning) are described in detail. Pre-processing includes choosing the device brand/model, placement of the device, algorithms/thresholds, and the number of days participants were required to wear the devices. Processing activities involve applying thresholds/algorithms to the data. Finally, post-processing includes data cleaning. The minimum number of days to be validated to correctly estimate weekly averages was specific to age and measurement. By summarizing and describing the methodological decisions and analysis protocol, we hope to contribute to the design and analysis of accelerometer data in future studies in similar research contexts.

## Resumo

Este estudo descreve as principais decisões metodológicas e suas justificativas para a coleta, processamento e limpeza/análise de dados de acelerometria nas coortes de nascimento de Pelotas, Rio Grande do Sul, Brasil, exemplificando como a pesquisa usando sensores de movimento pode ser realizada em países de renda média. Trata-se de uma pesquisa descritiva utilizando dados brutos do acelerômetro obtidos de cinco coortes brasileiras de base populacional. A amostra foi composta por dados de 32.963 indivíduos. A coleta de dados (decisões de pré-processamento), processamento (critérios para executar a análise) e as decisões de pós-processamento (limpeza de dados) foram detalhadas. O pré-processamento inclui a escolha da marca/modelo do dispositivo, posicionamento do dispositivo, algoritmos/limites e o número de dias que os participantes foram solicitados a usar os dispositivos. Processamento de dados envolve a aplicação de limites dos algoritmos. Finalmente, o pós-processamento inclui a limpeza de dados. O número mínimo de dias a serem validados para estimar corretamente as médias semanais foi específico para cada idade e tipo de medida. Ao resumir e descrever as decisões metodológicas e protocolo de análise, espera-se que o presente estudo contribua para o desenho de futuros trabalhos que busquem analisar dados do acelerômetro em contextos semelhantes.

## Resumen

Este estudio describe las principales decisiones metodológicas y sus justificaciones para la recopilación, procesamiento y limpieza/análisis de datos de acelerometría en las cohortes de nacimiento de Pelotas, Rio Grande do Sul, Brasil, ejemplificando cómo la investigación que utiliza sensores de movimiento puede llevarse a cabo en países de ingresos medianos. Se trata de una investigación descriptiva que utiliza datos sin procesar del acelerómetro obtenidos de cinco cohortes brasileñas de base poblacional. La muestra consistió en datos de 32.963 individuos. Se detalló la recopilación de datos (decisiones preprocesamiento), el procesamiento (criterios para realizar el análisis) y las decisiones posprocesamiento (limpieza de datos). El preprocesamiento incluye la elección de la marca/modelo del dispositivo, el posicionamiento del dispositivo, los algoritmos/límites y la cantidad de días que se les pidió a los participantes que usaran los dispositivos. El procesamiento de datos implica la aplicación de límites de los algoritmos. Por último, el posprocesamiento incluye la limpieza de datos. El número mínimo de días de validez para estimar correctamente los promedios semanales fue específico para cada edad y tipo de medida. Al resumir y describir las decisiones metodológicas y el protocolo de análisis, se espera que este estudio contribuya a futuros trabajos que busquen analizar los datos del acelerómetro en contextos similares.

## Introduction

Physical inactivity is responsible for > 5 million deaths per year worldwide ^1^. Sedentary behavior is associated with cardiovascular diseases, type 2 diabetes, and cancer mortality ^2^. Further, adequate sleep quality and duration are related to positive mental health and decreased risk of chronic diseases ^3^. Accurately measuring movement behaviors is important in public health to develop evidence-based guidelines. Globally, 86% of premature deaths due to noncommunicable diseases take place in low- and middle-income countries, highlighting the need to promote accurate measurement of movement behaviors in these settings ^4^. However, most accelerometry data from large-scale population studies come from high-income countries (e.g., UK Biobank, National Health and Nutrition Examination Survey, Rotterdam Study, International Children’s Accelerometry Database) ^5^ ^,^ ^6^ ^,^ ^7^ ^,^ ^8^. In this context, Pelotas (Rio Grande do Sul State), a middle-sized city in southern Brazil, is a rare example of accelerometer data collection in population-based studies in the Global South. The city hosts five population-based cohort studies (four birth cohorts and one older adult cohort).

There are two ways to analyze accelerometer data: traditional counts - post-filtered accelerometer values, with filters usually patented by companies - and raw data, which is the primary signal recorded by devices and expressed in gravitational acceleration. The Pelotas cohorts ^9^ ^,^ ^10^ ^,^ ^11^ ^,^ ^12^ ^,^ ^13^ pioneered using raw accelerometry data analyses, a methodological decision that potentially improves accuracy, transparency, and data harmonization ^14^ ^,^ ^15^. To date, researchers and collaborators of the Pelotas cohorts have published 33 studies using accelerometer data, from which nine used count-based analysis ^16^ ^,^ ^17^ ^,^ ^18^ ^,^ ^19^ ^,^ ^20^ ^,^ ^21^ ^,^ ^22^ ^,^ ^23^ ^,^ ^24^ and 24 used raw data analysis. Among the latter ^25^ ^,^ ^26^ ^,^ ^27^ ^,^ ^28^ ^,^ ^29^ ^,^ ^30^ ^,^ ^31^ ^,^ ^32^ ^,^ ^33^ ^,^ ^34^ ^,^ ^35^ ^,^ ^36^ ^,^ ^37^ ^,^ ^38^ ^,^ ^39^ ^,^ ^40^ ^,^ ^41^ ^,^ ^42^ ^,^ ^43^ ^,^ ^44^ ^,^ ^45^ ^,^ ^46^ ^,^ ^47^ ^,^ ^48^, most (54.2%) investigated the association of accelerometer-derived variables (e.g., overall physical activity, moderate to vigorous intensity physical activity [MVPA], etc.) with health outcomes, such as cognitive performance ^34^, falls ^28^, pulse wave velocity ^35^, use of medicines ^25^, sleep ^47^, child neurodevelopment ^38^, bone mineral density ^26^ ^,^ ^29^, body composition ^45^, cardiometabolic outcomes ^41^, preterm birth ^33^, and all-cause mortality ^27^. In addition, 29.2% were descriptive studies ^30^ ^,^ ^31^ ^,^ ^37^ ^,^ ^42^ ^,^ ^43^ ^,^ ^46^ ^,^ ^48^, and 16.7% were methodological ^32^ ^,^ ^36^ ^,^ ^39^ ^,^ ^44^. Most publications used physical activity variables such as overall physical activity (expressed in mg) (66.7%) and MVPA (66.7%). Light-intensity physical activity was used in 25% of studies, sedentary time in 12.5%, and sleep variables in only 8.3%. Supplementary Material 1 (https://cadernos.ensp.fiocruz.br/static//arquivo/suppl-e00011724_4052.pdf) presents detailed information about Pelotas publications using accelerometry in these cohorts.

All raw data analyses from the Pelotas cohort studies were conducted using the GGIR R package (https://cran.r-project.org/web/packages/GGIR/vignettes/GGIR.html) ^14^, which represented an advance in accelerometry, providing transparency in data cleaning/analysis. However, processing raw accelerometer data is highly complex in terms of software and code management, and computational limitations remain a challenge in contexts with limited research funding.

Over the course of more than a decade of data cleaning/analysis and collection, numerous updates have been made in accelerometer data processing ^14^. For example, in earlier datasets, the absence of sleep duration could affect the accuracy of calculating the total time spent on each intensity of physical activity. Additionally, decisions about the valid time of use considered in each cohort did not follow the same procedures, making it difficult to compare data across multiple waves.

Data collection protocols (pre-processing decisions), statistical analysis, and software features have generally been used by previous studies ^14^ ^,^ ^15^ ^,^ ^49^. However, the literature lacks information about data cleaning and harmonization issues that are also relevant and demand standardized procedures. In 2020/2021, all of Pelotas cohort’s accelerometer data files were reanalyzed, and methodological decisions and analyses protocols were summarized to improve transparency, comparability and aid future studies in similar research contexts. This study describes key methodological decisions and their justifications for accelerometer data collection, processing, and analysis in Pelotas cohorts.

## Methods

### Design and participants

**Figure 1:**
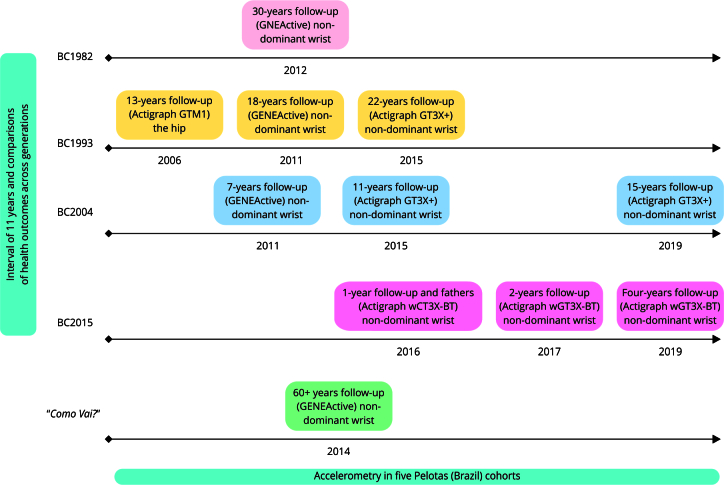
Follow-ups with accelerometer data in each Pelotas (Brazil) cohort.

Figure 1 presents the Pelotas cohorts with accelerometer data.

BC: birth cohort.

The first Pelotas birth cohort began in 1982 as a perinatal survey of all live births in the city’s maternity hospitals whose mothers lived in the urban area. With additional funding, the researchers decided to follow up the children at several points across their lifespan ^9^. The first accelerometer measurement from the 1982 cohort was conducted in 2012 (when participants were about 30 years old) using the GENEActive (https://activinsights.com/digital-health-technologies/professional-wearables/geneactiv/) device on the non-dominant wrist. Participants who visited the research clinic on Mondays, Tuesdays, or Wednesdays had the device collected the following Monday. Those who visited the clinic on Thursdays, Fridays, or Saturdays had the accelerometer collected the following Wednesday ^31^. This approach ensured all participants had four to seven free-living days, including at least one weekend day ^31^. More details about this protocol can be found elsewhere ^31^. The 1982 cohort is currently processing data from a new follow-up visit at the age of 40 using the Actigraph device.

The second cohort study included live births in 1993 and followed the same methodology of the 1982 study. This new cohort was designed to enable comparisons of health outcomes across generations (between cohorts). Detailed information about the 1993 birth cohort is available elsewhere ^10^. Regarding accelerometry, in the chronological timeline of Pelotas cohorts, 1993 was the first Pelotas to collect physical activity data as an objective measure in a 13-year-old sub-sample. However, this first data collection presented three differences compared to the next follow-ups: the use of a uniaxial accelerometer (GTM1, Actigraph, https://theactigraph.com/), the placement of the device on the hip, and the reduced sample size. We opted not to address this follow-up in detail in our study due to these substantial methodological differences. The first accelerometer measurement, including all cohort participants, was carried out in 2011 (when participants were 18 years old), following the same protocol used in the 1982 birth cohort ^31^. In 2015, the GT3X+ (Actigraph, https://theactigraph.com/) device was used, and the protocol was changed from four-to-seven days to seven days for all participants at the 22-year follow-up ^10^. This new protocol was possible due to the purchase of new accelerometers, increasing the number of devices available. The 1993 birth cohort is currently conducting a new follow-up at 30 years.

The third Pelotas (Brazil) birth cohort started in 2004 (the 2004 birth cohort) ^12^. It was the first Pelotas cohort to objectively measure physical activity in children. The first accelerometer measurement in this cohort was conducted in 2010 (at six years of age) following the first-adopted protocol (GENEActive for four-to-seven days) ^31^. In 2015 (when participants were 11 years old), the new protocol (Actigraph GT3x+ for seven days) was used. An additional follow-up occurred at 15 years of age in 2019, but was interrupted by the COVID-19 pandemic, with data being collected for approximately 50% of the cohort. Data from the 18-year follow-up is currently being processed.

The most recent Pelotas birth cohort began in 2015 and has two main additional characteristics. The first was the inclusion of baseline measurements during the pregnancy period, and the second was a specific focus on physical activity ^11^. In this cohort, mothers from all liveborn infants were invited to wear an accelerometer during pregnancy (between 16 and 24 months) and when their children (i.e., the cohort participants) were two years old. Accelerometry data was also collected from fathers when cohort participants were one year old. Data on mothers and fathers were collected for seven days on the non-dominant wrist using Actigraph. At ages one and two, the children wore the monitors for four days. This decision was based on a previous study assessing required time of use, placement (wrist or ankle), and bracelet material for very young children ^50^.

In addition to these, the fifth cohort in Pelotas focuses on older adults. The “*Como vai?*” *Estudo Longitudinal de Saúde do Idoso* (*“How’s it going?” Longitudinal Study of Elderly Health*) study ^13^ began in 2014 as a population-based survey designed to be representative of the community-dwelling population of 60+ years of age residing in the urban area of the city. After three years, the study became a cohort with follow-ups in 2017, 2019, and 2022. Accelerometer data are available only at baseline and was obtained using the GENEActive device on the non-dominant wrist for seven days.

### Data summarization

We chose to present the processing in the results section using raw data provided by a triaxial accelerometer, since count-based and uniaxial accelerometers were analyzed in a different process (using the companies’ software). Most analyses carried out in the Pelotas cohorts use raw data and triaxial devices (GENEActive and GT3X+). Raw data refers to all raw acceleration signals measured in mg/unit of time. The research team is responsible for data cleaning and interpreting metrics.

### Ethics

All follow-ups were approved by the Ethics Research Committee of the School of Medicine or School of Physical Education Ethics Committee, Federal University of Pelotas. All participants were asked to fill an informed consent form. The protocol numbers of each approval were: n. 16/12 (1982 birth cohort at 30 years), n. 05/2011 and 1.250.366 (1993 birth cohort at 18 and 22 years); n. 35/10, 889.753/CAAE: 38013414.9.0000.5317 and 3.554.667/CAAE: 20183419.1.0000.5317 (2004 birth cohort at seven, 11 and 15 years), 26746414.5.0000.5313 (2015 birth cohort - pregnancy to four years of age) and 201324538513.1.0000.5317 (“*Como vai?*” study).

## Results

### Pre-processing methodological decisions - data collection

The pre-processing phase of accelerometer data collection demands several methodological decisions, including device brand, placement, time of use, seasonality of data collection, and other aspects. Although this article does not focus on data collection, some aspects should be highlighted.

Unlike in high-income countries, we cannot rely on mail services in Brazil to send/return accelerometers due to costs, logistics, limited quality of service and safety. Therefore, the participants of the Pelotas cohorts have the accelerometer placed upon their visit to the research clinic, where they answer questionnaires and are examined (e.g., DXA, Bod Pod, blood sample, etc.). A research assistant collects the accelerometer after the data collection period for a given participant has elapsed. This procedure generates additional costs compared to mail services, but improves compliance. When the device is returned to the research clinic, a cohort staff downloads the data and performs a visual inspection as a first quality control procedure. If the accelerometer is used for less than half of the expected time, a new contact is made to try a new data collection.

The use of wrist-worn accelerometers is not usual, with hip-based measures being more common in the literature. Nevertheless, the wrist placement was chosen mainly due to higher compliance than hip placement ^51^ ^,^ ^52^. Additionally, an increasing number of cut-off points and algorithms are being developed for wrist-based accelerometry to define time spent in different intensities in each age. Lastly, sleep measures are better detected when the device is wrist-worn ^53^.

### Processing methodological decisions

To implement all filtering and processing required to analyze raw data, we used the GGIR R package, a free tool available for the R program (http://www.r-project.org) ^14^, which works in five steps. The first generates metadata for the following stages: calculating metrics, performing an auto-calibration process, and detecting non-wear time. The second step incorporates non-wear time and summarizes metrics based on acceleration, providing quality data plot for each individual, as well as daily and weekly reports of physical activity measures. The third step detects periods of sustained inactivity. The fourth step detects sleep periods and generates a daily and weekly report for sleep measures. The last step merges physical activity (step two) and sleep (step four) reports ^14^. In the GGIR package, there are two options to define a one-day period: (a) midnight to midnight, and (b) waking up to waking up. Our standard analysis protocol used option (b), but option (a) could be used for specific analysis (mainly in compositional analyses using an exact cycle of 24 hours). GGIR enables customizing the criterion to consider a day valid (minimum time per day to consider a day period in the analysis), but we used the default criterion (16 hours). Supplementary Material 2 (https://cadernos.ensp.fiocruz.br/static//arquivo/suppl-e00011724_4052.pdf) brings the thresholds for MVPA and sedentary behavior proposed by Hildebrand et al. ^54^ ^,^ ^55^, sleep algorithms proposed by van Hees et al. ^56^ ^,^ ^57^, and the code we used in our analyses. The thresholds to define MVPA varied according to the participants’ age in each follow-up. Also, these thresholds influence final measures but not the processing protocol, which should be equal between samples in order to compare them. Most of our analytical decisions reflect our interest in both physical activity and sleep. Therefore, the final datasets were generated by applying cleaning parameters considering both outcomes. Steps two, four and five, respectively, generate .csv files with variables for physical activity, sleep, and physical activity combined with sleep.

### Post-processing methodological decisions - data cleaning

**Figure 2:**
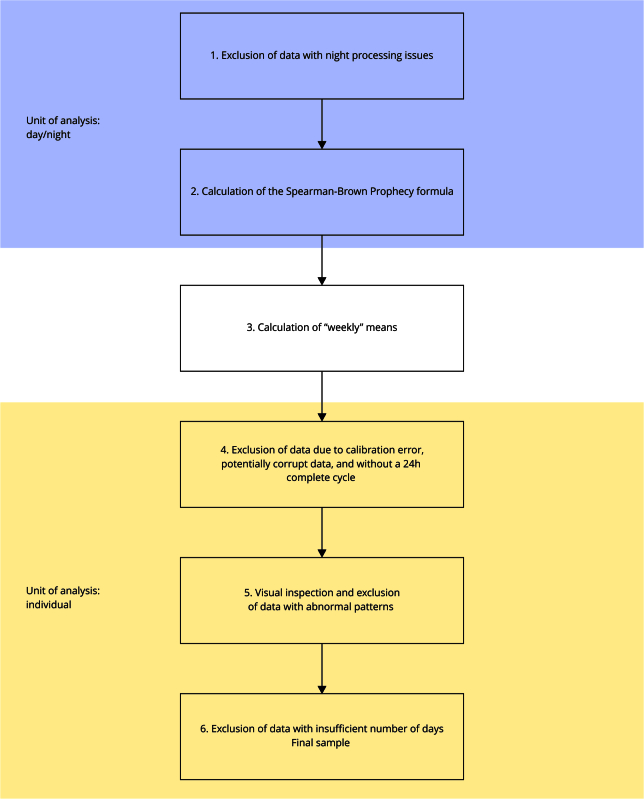
Summary of the accelerometer data analysis protocol in the Pelotas (Brazil) cohorts.

Figure 2 summarizes the steps of data processing/analysis from cohorts after obtaining .csv files for each individual in the sample. Considering the follow-ups of the 2015 birth cohort at one, two, and four years of age, no sleep information was generated from the accelerometer data, as there is currently no validated algorithm to analyze sleep at these ages when using raw data.

We started cleaning the data after importing results generated by GGIR (stored in .csv files) to statistical software (Stata, SPSS, R, etc.). Step one uses the day summary output - that is, the datasets with each line representing estimates for each day/night (GGIR generates this file under the name “daysummary.csv”). This step excludes day or night periods that could present any sleep detection problem affecting the complete day estimates from step five (using the variable “cleancode” in GGIR). Following these exclusions, with the day/night unit of analysis, step two explores the possibilities for a number of days considered valid to estimate a weekly average, using the Spearman-Brown Prophecy formula (described below). 

In step three, we calculate the weekly averages of variables, and in step four, we exclude data that fail to achieve certain quality parameters. These exclusions include data with a calibration error higher than 0.02, data without a complete 24-hour cycle, and potentially corrupted data (fraction of 15-minute windows for which acceleration in one of the three axes was close to the maximum for at least 80% of the time, clipping score = 1).

The fifth step is to visually inspect the data using plots generated for each participant and stored as a .pdf file. These plots contain the acceleration of each device to identify possible unexpected patterns that may not have been identified in previous steps. This step is mostly manual and subjective, and should be carried out jointly with the previous steps to exclude all problematic data. We are interested in identifying plots with very high acceleration values, very low variation or acceleration, or very far from zero during the data collection period for each individual (Supplementary Material 3 − https://cadernos.ensp.fiocruz.br/static//arquivo/suppl-e00011724_4052.pdf − includes examples of usual and abnormal data in plots).

Finally, step six refers to the results from the Spearman-Brown Prophecy formula (Table 1 and text below). Supplementary Material 4 (https://cadernos.ensp.fiocruz.br/static//arquivo/suppl-e00011724_4052.pdf) shows the definitions of variables presented in Table 1. After considering data quality and number of exclusions, the number of valid days is chosen and applied for the entire follow-up. Consequently, individuals with fewer days than the minimum number required are excluded. These requirements could vary depending on population, and the Spearman-Brown Prophecy formula varies from 0 to 1, with higher values indicating better intraclass correlation.

**Table 1:** Spearman-Brown Prophecy formula to choose the number of days for each follow-up.

Cohort	Follow-up	Variable	Number of days					
			1	2	3	4	5	6
1982 Birth cohort	30 years (GENEActive)	MVPA	0.48	0.65	0.73	0.78	0.82	0.84
		MVPA 5 minutes	0.44	0.61	0.70	0.76	0.80	0.82
		TST	0.22	0.36	0.46	0.53	0.58	0.63
1993 Birth cohort	18 years (GENEActive)	MVPA	0.48	0.65	0.73	0.78	0.82	0.85
		MVPA 5 minutes	0.45	0.62	0.71	0.76	0.80	0.83
		TST	0.13	0.23	0.31	0.37	0.43	0.47
	22 years (Actigraph)	MVPA	0.55	0.71	0.78	0.83	0.86	0.88
		MVPA 5 minutes	0.47	0.64	0.72	0.78	0.81	0.84
		TST	0.16	0.28	0.37	0.44	0.45	0.54
2004 Birth cohort	6 years (GENEActive)	MVPA	0.49	0.66	0.74	0.80	0.83	0.85
		MVPA 5 minutes	0.42	0.59	0.68	0.74	0.78	0.81
		TST	0.19	0.32	0.41	0.48	0.54	0.58
	11 years (Actigraph)	MVPA	0.52	0.69	0.76	0.81	0.84	0.86
		MVPA 5 minutes	0.46	0.63	0.72	0.77	0.81	0.84
		TST	0.05	0.10	0.14	0.18	0.21	0.25
	15 years (Actigraph)	MVPA	0.47	0.64	0.73	0.78	0.82	0.84
		MVPA 5 minutes	0.39	0.57	0.66	0.72	0.77	0.80
		TST	0.04	0.08	0.12	0.15	0.18	0.21
2015 Birth cohort	Pregnancy (mother) (Actigraph)	MVPA	0.61	0.76	0.83	0.86	0.89	0.90
		MVPA 5 minutes	0.43	0.60	0.69	0.75	0.79	0.82
		TST	0.21	0.35	0.45	0.52	0.58	0.62
	2 years (mother) (Actigraph)	MVPA	0.63	0.77	0.84	0.87	0.89	0.91
		MVPA 5 minutes	0.46	0.63	0.71	0.77	0.81	0.83
		TST	0.23	0.38	0.48	0.55	0.60	0.64
	1 year (father) (Actigraph)	MVPA	0.54	0.70	0.78	0.83	0.86	0.88
		MVPA 5 minutes	0.48	0.65	0.74	0.79	0.82	0.85
		TST	0.22	0.37	0.46	0.54	0.59	0.63
	4 years (children) (Actigraph)	ENMO *	0.44	0.61	0.70	0.76	0.80	0.83
“*Como Vai*?”	60+ years (GENEActive)	MVPA	0.76	0.87	0.92	0.93	0.95	0.96
		MVPA 5 minutes	0.52	0.68	0.76	0.81	0.84	0.87
		TST	0.46	0.63	0.72	0.77	0.81	0.83

MVPA: moderate to vigorous physical activity; TST: total sleep time.

Note: dark spots represents stronger intraclass correlation values, while light spots represents weaker intraclass correlation values.

* For the 2015 Birth cohort (four years), only overall physical activity (ENMO) is calculated due to lack of adequate thresholds/algorithms with raw data and the placement of the device for this age.

**Figure 3:**
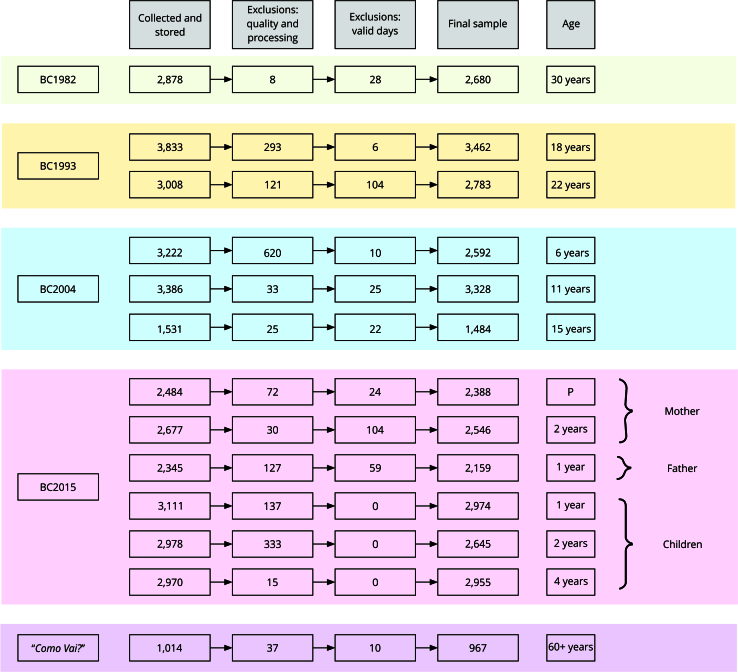
Flowchart summarizing accelerometer data processing and analysis in each cohort follow-up.

BC: birth cohort; P: pregnancy.

Figure 3 shows the number of individuals with accelerometer data analyzed in each follow-up of each cohort, as well as the number of exclusions related to data quality (individual identification problems, processing problems, calibration error, unexpected high acceleration, visual inspection, and sleep detection issues) and number of valid days. In total, 13 follow-ups from five Pelotas cohorts (four birth cohorts and one older adults’ cohort) were analyzed, resulting in 32,963 files. Detailed flowcharts of each follow-up are presented in Supplementary Material 5 (https://cadernos.ensp.fiocruz.br/static//arquivo/suppl-e00011724_4052.pdf).

Table 1 shows the Spearman-Brown Prophecy formula values used to decide the number of valid days in each follow-up for MVPA with no bouts, MVPA with 5-minute bouts, and total sleep time. The analysis was performed from one to six days to identify the minimum number of days considered valid to include individuals in weekly estimates. For the 2015 birth cohort follow-ups at one, two, and four years of age, the analysis was only carried out with overall physical activity (ENMO mean in 24h) due to the current absence of established MVPA cut-offs and sleep algorithm for raw data in these ages. This analysis was not possible for the first two follow-ups of the 2015 Birth cohort, when children wore the devices for only four days (as aforementioned). The decision of four days of data collection was based on a pilot study with children from 9 to 16 months (not participants of the cohort) to define the number of days needed to estimate overall physical activity at these ages (one day without measurement to avoid reactivity, 48h of measurement and one day to return the device to the team) ^50^. With these results, we can identify that the threshold for a number of valid days could be sample-specific and, ideally, the Spearman-Brown Prophecy formula should be used to determine a minimum of days according to the age of the sample and the main measure of the analysis (focusing on overall physical activity, MVPA or sleep).

For the other follow-ups, there is a clear pattern showing that, for MVPA with no bouts, two or three days were sufficient to reach the 0.7 threshold in the Spearman-Brown Prophecy formula (considered satisfactory to estimate global average) in most follow-ups. However, for variables with higher requirements such as MVPA 5-minute bouts, and especially for total sleep time, the number of days required to reach 0.7 in the Spearman-Brown Prophecy formula is higher.

After the abovementioned processes, final datasets (post exclusions) are shared with each cohort data manager containing a group of basic, interpretable variables (listed in Supplementary Material 4 - https://cadernos.ensp.fiocruz.br/static//arquivo/suppl-e00011724_4052.pdf), which will be available for empirical investigations involving physical activity and/or sleep. Additionally, the original .csv files with all information provided from GGIR (reports for steps two, four and five) are stored to facilitate generating specific variables not included in usual datasets, if necessary.

Lastly, in the Pelotas studies using accelerometry, we compared the original sample to individuals who presented valid accelerometer data according to gender, education, and socioeconomic position. We performed this to identify possible biases in accelerometer data, emphasizing caution when interpreting results. In the Pelotas cohorts, accelerometer samples were very similar to the original samples, ensuring the characteristics of population-based studies ^31^ ^,^ ^39^ ^,^ ^42^.

## Discussion

The Pelotas cohorts contain a robust and unique amount of accelerometer data for a middle-income country, with pre-processing data files for 32,963 individuals. In addition to publications regarding accelerometer data processing ^14^ ^,^ ^15^ ^,^ ^49^, the documentation process providing a big picture of methodological decisions can help other studies plan accelerometer data collection and analysis. Here, we present relevant topics to consider when collecting device-measured physical activity data in middle-income countries, such as the different thresholds to be used to define the number of valid data depending on the participants’ age and variables of interest (for example, MVPA versus total sleep time).

In Brazil, studies involving accelerometers face significant challenges, such as the logistic complexity of placing and collecting the monitors. Furthermore, data processing requires training teams and professionals with specific technical knowledge of the model, software, and data cleaning/analysis ^36^. Additionally, the high cost, short lifespan, failures, and losses of monitors can compromise the sustainability and continuity of the research, resulting in incomparable data due to different algorithms used by manufacturers and device brands ^58^ ^,^ ^59^. However, initiatives such as the Pelotas cohorts stand out for overcoming these difficulties, performing analysis of raw data using open-source software and offering more transparency and comparability between studies ^49^ ^,^ ^60^.

Accelerometer studies require extensive planning due to the devices’ high cost and the complexity of the logistics involved. Although the need for funding and planning to collect accelerometer data is high, most studies only use data of weekly averages of MVPA or sleep. At the same time, more detailed and creative research questions remain necessary. For example, out of 25 published papers by the Pelotas cohorts with raw accelerometer data, only two explored the averages of physical activity according to time of day ^47^ ^,^ ^48^. Given the availability of many measurements in three different axes by second during one week for each participant, it is clear these data could be further explored. Current technology enables exploring acceleration patterns during the day, different bouts of physical activity, intensity gradients, other aspects of sleep besides duration (e.g., efficiency, fragmentation index), rhythmicity (e.g., intradaily variability and interdaily stability), and compositional analysis or averages in weekdays and weekends.

Accelerometry provides no information about domains of physical activity. Thus, it is essential to measure physical activity using questionnaires to complement accelerometer data. Some studies exploring the physical activity paradox indicate that the association with health outcomes could differ depending on the domain of physical activity, with work-related activities being the main representative of possible risk to diseases or absence of association ^61^ ^,^ ^62^ ^,^ ^63^. Generally, accelerometers are more precise than questionnaires, as they are not based on the perceptions, understanding, and values of individuals ^54^ ^,^ ^56^. In turn, questionnaires remain relevant when analyzing social well-being or psychological effects of physical activity. Regarding physical activity promotion, most interventions focus on leisure-time and/or commuting physical activity - for example, public spaces to work out, green areas availability, sport and school interventions, walkability, etc.

Methodological decisions could interference in the results of accelerometry. Currently, we still have a lack of comparability because each research group follows a different protocol for pre-processing, processing, and post-processing. In our study, we tried to clarify all relevant steps in accelerometry analysis. Further, data cleaning is a detailed process, including visual inspection and data exclusions with calibration errors, non-human movement, etc. Finally, our analysis regarding the minimum number of days to be considered valid showed that this number is sample-specific. For example, while older individuals presented a more regular pattern throughout the days, children and adolescents presented a more irregular pattern, requesting more days to estimate better daily week averages. This number of days will directly influence the final sample size and consequent statistical power of analysis. Thus, we recommend running an analysis such as the Spearman-Brown Prophecy formula for each sample evaluated.

This study presents some limitations. First, the definition of sedentary behavior includes a postural measure, which most accelerometers do not collect. Thus, our sedentary time variable is based only on very low acceleration. Although many other studies use this measure as a proxy of sedentary behavior, it should be confirmed by well-conducted validation studies, which are currently lacking. Also, most studies with accelerometers used the period from midnight to midnight to define a 24-hour cycle. However, the Pelotas cohorts opted to use the wake-to-wake cycle. This decision sometimes generates a problem of days with more or less than 24 hours, but it is likely to better reflect the individuals’ routines.

Our study also has considerable strengths. We present methodological decisions for one of the largest datasets in a middle-income country, including five population-based cohorts with accelerometer data at multiple time points (except for the older people cohort, which collected accelerometer data only at baseline) and in different generations and life stages. Additionally, the round of analysis using the same analysis protocol for all follow-ups improves comparability within and between cohorts and makes data processing transparent, contributing to comparisons with external studies. Finally, because initial data from cohorts were processed over 10 years ago, many follow-ups did not include sleep, light physical activity, sedentary behavior, or rhythm variables that were inserted with the new rounds of analysis.

## Conclusions

This study documented the methodological decisions for accelerometer data collection and processing in five Pelotas cohorts. Different thresholds were presented to define the number of valid days depending on age and variables under consideration. We also described the stages of data cleaning and harmonizing, which are usually not well reported in the literature. By summarizing and describing the methodological decisions and analysis protocol, we hope to contribute to the design and analysis of accelerometer data in future studies in a similar research context.
