# Supplementary material for: Lessons from 13 years of accelerometry measurements in five Brazilian cohorts: methodological aspects
Source: Cad Saude Publica. 2025 Apr 11;41(3):e00011724. doi: 10.1590/0102-311XEN011724 (PMC11996192; doi:10.1590/0102-311XEN011724)

Supplementary Materials

Supplementary Material 1: Review of literature – Pelotas cohort accelerometer studies

a) Method

We carried out a scoping review to identify the analysis protocol being applied and the main results they provide in the Pelotas cohorts. The research question was the following: "What are the characteristics of all studies from the Pelotas Cohorts, including accelerometer measures?".

Regarding the inclusion and exclusion of articles, we searched for all studies using accelerometer data in Pelotas cohorts, descriptive and methodological manuscripts, and studies assessing the association of physical activity objectively measured with some health outcome. Because accelerometry in the cohorts are focused on raw data, we excluded from the main data summarization nine count-based studies (subsample at 13 years follow-up from BC1993). However, this set of results is presented in Supplementary material 6.

The search was carried out in April of 2022 in following databases: PubMed, Lilacs and Scopus. The search strategy combines the following descriptors: ("accelerometer" OR "accelerometry" OR "objective measure" OR "objectively measured") AND ("cohort" OR "como vai?" OR "longitudinal" OR "prospective" OR "older adults" OR "elderly") AND ("Pelotas" OR "South" OR "Southern"). The complete search strategy and details/adaptations for each database is presented below, in Supplementary material 1-B.

The data selection and extraction were carried by two researchers simultaneously and in case of doubt about inclusion, a third researcher was consulted. The stages of review were the following: a) selection of titles; b) selection of abstracts; c) selection of complete text; d) reading of reference lists; e) data extraction and f) summarization of results. The results extracted were the following: author, year, main aim (categorized as descriptive, association with health aspect or methodological), sample (total participants, follow-up/age and cohort), device, PA measures, sleep measures and main results.

Data extracted included the following information: author, year, aim, type of article (methodological, descriptive, association), sample size, age of participants, model of accelerometer, accelerometer measures, main results, and any relevant observation in the studies.

Lastly, data were summarized in descriptive tables (one for count-based measures and another for raw data) and a graph using only raw-based measures, including percentages of studies in each cohort, the device model, the paper's aim, and the collected measures.

b) Strategy search

	**Accelerometer**		**Cohort**		**Pelotas**
Pubmed (Title/abstract)	"accelerometer" OR "accelerometry" OR "objective measure" OR "objectively measured"	**AND**	"cohort" OR "como vai?" OR "longitudinal" OR "prospective" OR "older adults" OR "elderly"	**AND**	"Pelotas" OR "South" OR "Southern"
Scopus (Title/abstract/keywords)	"accelerometer" OR "accelerometry" OR "objective measure" OR "objectively measured"	**AND**	"cohort" OR "como vai?" OR "longitudinal" OR "prospective" OR "older adults" OR "elderly"	**AND**	"Pelotas" OR "South" OR "Southern"
Lilacs	"acelerometro" OR "acelerometros" OR "acelerometria"	**AND**	"coorte" OR "coortes" OR "longitudinal" OR "prospectivo" OR "idosos"	**AND**	"Pelotas" OR "Sul"

c) Results

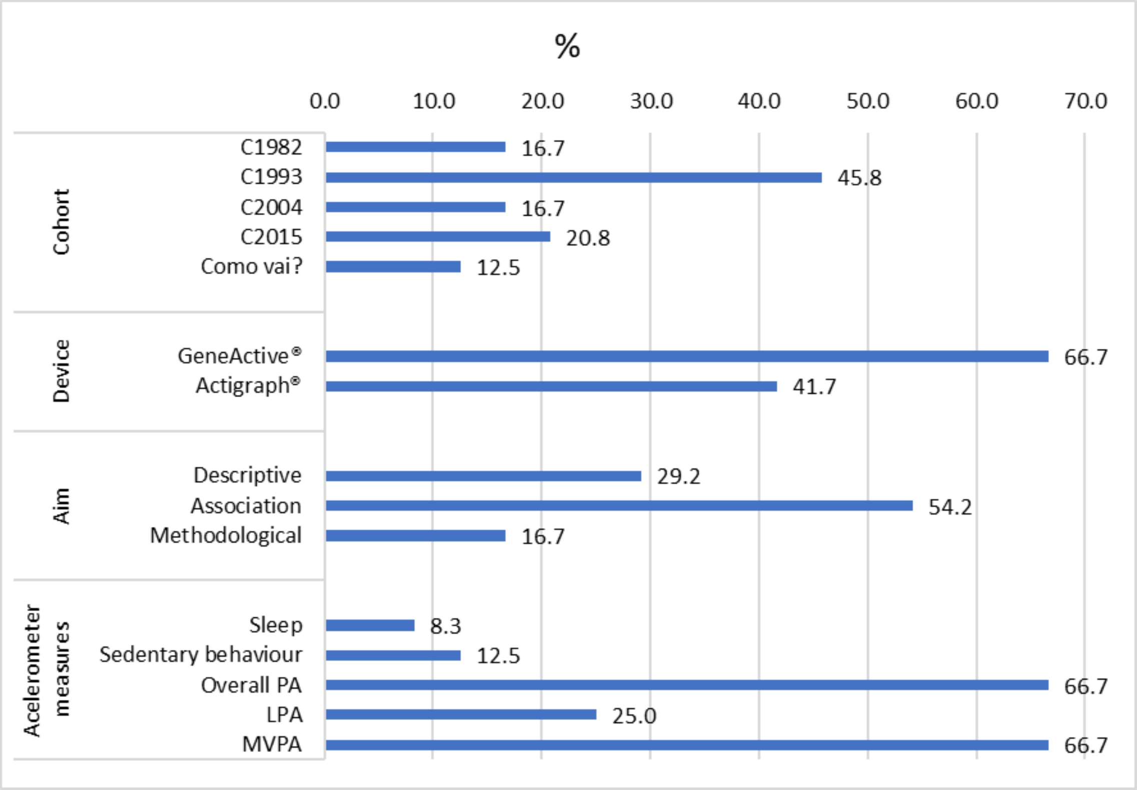

%
0.0
10.0
20.0
30.0
40.0
50.0
60.0
70.0
Cohort
C1982
16.7
C1993
45.8
C2004
16.7
C2015
20.8
Como vai?
12.5
Device
GeneActive®
66.7
Actigraph®
41.7
Aim
Descriptive
29.2
Association
54.2
Methodological
16.7
Accelerometer measures
Sleep
8.3
Sedentary behaviour
12.5
Overall PA
66.7
LPA
25.0
MVPA
66.7

d) Summarized information from articles with C1993 subsample (GTM1 device used on the hip).

Autor	Year	Aim	Group	Sample	Device	PA measures	Main results
Reichert et al. [16]	2009	A methodological model for collecting high-quality data on physical activity in developing settings-the experience of the 1993 Pelotas (Brazil) Birth Cohort study	Methodological	C1993 (subsample, 13y), N = 457	Actigraph (GTM1)	Number of days the Actigraph had registered time above 600 min; Time spent in moderateintensity activities (2000 to 4999cpm); Time spent in vigorousintensity activities (≥ 5000cpm)	High response rate (90%). Main challenges to carri out the follow-up were described in the text
Gigante et al. [17]	2010	To describe and compare two methods of energy intake assessment and one measure of energy expenditure applied in adolescents from a birth cohort	Methodological	C1993 (subsample, 13y), N = 183	Actigraph (GTM1)	Energy expenditure (Trost)	Average energy expenditure estimated by accelerometers was 2,356kcal (± 460) and the median was 2,303kcal (inter-quartile range – IQR = 548). The mean difference between FFQ and accelerometer (357 ± 968cal/day) was lower than the difference of both food-frequency questionnaire and 24h recall methods.
Hallal et al. [18]	2011	To explore cross-sectional and longitudinal associations between self-reported and accelerometry-based physical activity (PA) and blood pressure (BP) between 11 and 14 years of age.	Associations with health variable	C1993 (subsample, 13y), N = 427	Actigraph (GTM1)	Minutes of MVPa in terciles	PA measured by accelerometry at 12 years, but not questionnaire-derived PA, was inversely associated with diastolic BP at 14 years of age in fully adjusted models. Those who exceeded the 300-minutes PA threshold at all 3 visits had a 2.6 mmHg lower mean increase in DBP from 11 to 14 years compared with those classified below the threshold in all visits
Wells et al. [19]	2011	To investigate differences between firstborn and later-born individuals in early growth patterns, body composition, and blood pressure in Brazilian adolescents	Associations with health variable	C1993 (subsample, 13y), N = 453	Actigraph (GTM1)	Accelerometry (1,000 counts); Accelerometry (minutes/week MVPA)	In adolescence, firstborns had significantly greater height and blood pressure and a lower activity level. The

							difference in systolic blood pressure could be attributed to variability in early growth and that in diastolic blood pressure to reduced physical activity.
Hallal et al. [20]	2012	To evaluate the effects of prenatal, infancy and childhood weight and length/height gains on objectively-measured physical activity (PA) in adolescence.	Associations with health variable	C1993 (subsample, 13y), N = 4 57	Actigraph (GTM1)	Sedentary (0-100 counts); Light (101-2000 counts); Moderate (2001-5000 counts); Vigorous (5001-8000 counts); Very vigorous (> 8000 counts); % active (≥ 60 min/day of moderate-to-vigorous physical activity)	Weight and length/height trajectories in infancy and childhood were similar between those classified as active or inactive at 13.3 years. However, those classified as inactive were heavier and taller at all ages; differences were statistically significant only in terms of length at three, six and 12 months.
Hallal et al. [21]	2012	To investigate the cross-sectional and prospective associations between physical activity and body composition in adolescence	Associations with health variable	C1993 (subsample, 13y), N = 457	Actigraph (GTM1)	MVPA (minutes/week-1); % 5300 minutes/week-1	In fully-adjusted models, no significant cross-sectional or longitudinal associations were found between physical activity and body composition, neither in boys nor in girls. These null results were confirmed in the 511 individuals with accelerometry and deuterium data
Reichert et al. [22]	2012	To describe the patterns of objectively measured physical activity in Brazilian adolescents.	Descriptive	C1993 (subsample, 13y), N = 486	Actigraph (GTM1)	Total physical activity (cpm); ≥ 60 min·d^{-1} of MVPA; Time spent in MVPA	The mean times (in minutes) spent in sedentary, light, moderate, and vigorous activities were 962 (SD = 114), 176 (SD = 46), 36 (SD = 16), and 17 (SD = 10) min·d−1, respectively. More than 30% of the adolescents accumulated ≥ 60 min·d−1 of moderate-to-vigorous physical activity (MVPA). Physical activity

							intensity (counts per minute) and the prevalence of accumulation ≥ 60 min·d−1 of MVPA were higher among boys and in those who walked or cycled to school. The prevalence of accumulation > 60 min·d−1 of MVPA was 125% higher in those from lower economic levels compared with higher economic levels ($p < 0.001$).
Reichert et al. [23]	2012	To explore the association between objectively measured physical activity and body composition indices in 13-year old adolescents living in country undergoing rapid nutritional transition	Associations with health variable	C1993 (subsample, 13y), N = 457	Actigraph (GTM1)	Time spent in sedentary activities; Time spent in MPA; Time spent in VPA; Total counts; Counts per minute;% ≥ 60min/day of MVPA	A one minute/day increase in vigorous intensity physical activity was associated, on average, with a reduction of 0.15cm in waist circumference ($p = 0.007$) and 0.20mm in sum of skinfold thicknesses ($p = 0.02$). These associations were stronger in boys than in girls. Total activity and time spent in moderate intensity physical activities were not associated with any of the outcomes in the adjusted analyses. Vigorous physical activity appears to have the strongest association with body composition.
Hallal et al. [24]	2013	To compare physical activity measured by accelerometer and questionnaire against total (TEE) and physical activity energy expenditure (PAEE) estimated by DLW	Methodological	C1993 (subsample, 13y), N = 25	Actigraph (GTM1)	Time spent in sedentary activities; Time spent in MPA; Time spent in VPA; Time spent in MVPA	TEE varied from 1,265 to 4,143 kcal/day. It was positively correlated with physical activity (counts) estimated by accelerometry (rho = 0.57; $p = 0.003$). An increase of 10 minutes per day in moderate-to-vigorous intensity physical activity (MVPA) relates to an increase in TEE of 141

								kcal/day. PAEE was positively correlated with accelerometry (rho = 0.64; p = 0.007), but not with minutes per week of physical activity estimated by questionnaire (rho =0.30; p = 0.15). Physical activity by accelerometry explained 31% of the vssariability in TEE. By incorporating fat and fat-free mass in the model, we were able to explain 58% of the variability in TEE.

e) Summarized information from selected articles (device used on the non-dominant wrist).

AUTHOR-YEAR	AIM	GROUP	SAMPLE - DEVICE	MEASURES	MAIN RESULTS	OBSERVATIONS
Knuth, et al. [36] 2013	The aim of this study was to characterize the methodology of data collection on physical activity using accelerometry in two birth cohorts (2004 and 1993) in Pelotas, Rio Grande do Sul State, Brazil, at the 6-7 and 18-year follow-up visits, respectively.	Methodological	C1993, (18y), N = 4,106 C2004, (6,7y), N = 3,331 Genea and GENEActiv	-	The study characterizes the data collection methodology in more than 7,000 individuals and discusses issues in its implementation. It thus provides a methodological framework aimed at helping to plan future population-based studies with the use of such technology and to improve understanding of physical activity in the context of epidemiological studies.	The 2004 cohort collected 514 data with the Genea model, however, due to problems with the device's memory, difficulty with downloads, inadequate wristbands and fragility of the monitors, they were gradually replaced by the GENEActiv model, which was used until the end of follow-up.
Silva et al. [31] 2014	The aim of this study was to describe objectively measured overall physical activity and time spent in moderate-to-vigorous physical activity (MVPA) in individuals from the Pelotas (Brazil) birth cohorts, according to weight status, socioeconomic status (SES) and sex.	Descriptive	C1982, (30y), N = 2,876 C1993, (18y), N = 3,822 C2004,(7y), N = 3,331 Genea and GENEActiv	MVPA in 5- and 10-min bouts	Mean acceleration in the 1982 (mean age 30.2 years), 1993 (mean age 18.4 years) and 2004 (mean age 6.7 years) cohorts was 35 mg, 39 mg and 60 mg, respectively. Time spent in MVPA was 26 [95% confidence interval, 95%CI: 25; 27], 43 (95%CI: 42; 44) and 45 (95% CI 43; 46) min/d in the three cohorts, respectively, using 10-min bouts. Mean MVPA was on average 42% higher when using 5-min bouts. Males were more active than females and physical activity was inversely associated with age of the cohort and SES. Normal-weight individuals were more active than underweight, overweight and obese participants.	-
Esteban-Cornejo et al. [34] 2015	This study aimed to examine the prospective associations of physical activity at 11, 15, and 18 years of age with cognitive performance in young adulthood in a large birth cohort study from Brazil.	Association with health variable	C1993, (18y), N = 3.097 GENEActive	MVPA in 10-min bouts	At 11 years, participants in the middle tertile of self-reported physical activity presented a significantly higher cognitive performance score as compared with the lowest tertile. Physical activity at 15 yr of age was unrelated to cognitive performance at 18 yr. Self-reported physical activity was cross-sectionally positively associated with cognitive performance at 18 yr ($P < 0.001$). Data from objectively measured physical activity at 18 yr showed that those in the highest moderate-to-vigorous physical activity tertile presented lower	-

					cognitive performance scores at 18 yr as compared with those in the lowest tertile (-2.59; 95%CI: -3.41; -1.48). Analyses on changes in tertiles of physical activity showed that maintaining an intermediate physical activity level from 11 to 18 yr and from 15 to 18 yr was associated with a higher cognitive performance score of 2.31 (95%CI: 0.71; 3.91) and 1.84 score (95%CI: 0.25; 3.42), respectively.	
Horta et al. [35] 2015	To examine the associations between objectively measured physical activity and sedentary time with pulse wave velocity (PWV) in Brazilian young adults.	Association with health variable	C1982,(30y), N = 1,241 GENEActiv	Overall PA: ENMO vartiable provided in mg, MVPA in 10-min bouts, Sedentary behaviour	Complete data were available for 1241 individuals. PWV was significantly lower in the two highest quartiles of overall PA (0.26 m/s) compared with the lowest quartile. Participants in the highest quartile of sedentary time had 0.39 m/s higher PWV (95%CI: 0.20; 0.57) than those in the lowest quartile. Individuals achieving ≥30 min/day in MVPA had lower PWV (β = -0.35; 95%CI: -0.56; -0.14). Mutually adjusted analyses between MVPA and sedentary time and PWV changed the coefficients, although results from sedentary time remained more consistent. WC captured 44% of the association between MVPA and PWV. DBP explained 46% of the association between acceleration and PWV.	-
Bergmann et al. [25] 2016	Evaluate cross-sectional and longitudinal associations between physical activity, screen time, and use of medicines among adolescents from the 1993 Pelotas (Brazil) birth cohort study, followed at 11 (N = 4,452), 15 (N = 4,325), and 18 years of age (N = 4,106).	Association with health variable	C1993, (18y), N = 3,833 GENEActiv	Overall PA: ENMO vartiable provided in mg	One-third of adolescents had used at least one medicine in the previous 15 days and approximately 10% were on some continuous medication. Individuals in 3rd tercile of PA at 18y presented lower OR for use of medicines (OR = 0.44; 95%CI: 0.32; 0.62). This association was more evident in men R = 0.49) than in women (OR = 0.77)	-
Knuth et al. [37] 2017	To examine the longitudinal influences of early life social and biological indicators on objectively	Descriptive	C2004 (6y), N = 2.604 GENEActiv	Overall PA: ENMO vartiable provided in mg	Girls were less active than boys (β = -8.65mg; 95% CI: -10.0; -7.30). Higher socioeconomic position was related to lower activity levels (β = -9.69mg. 95%CI: -12.45; -6.93) and a similar	The first 10hours and the last 20 h of data in each raw accelerometer file were excluded as these were potential periods when the accelerometers would not be attached to the

	measured physical activity				association was found with maternal schooling. No associations were found with birthweight, type of delivery or preterm delivery.	participants
Ramires et al. [42] 2017	Describe objectively measured physical activity levels among older adults residents in a Southern city of Brazil.	Descriptive	Cohort Como Vai, (60y+), N = 971 GENEActiv	Overall PA: ENMO vartiable provided in mg, MVPA in 1,5 and10-min bouts, LPA (non-bouted)	Women spent on average more time on LPA (136.2 vs. 127.6 min per day). Men and women respectively accumulated, in average, 64.5 and 56.7 min per day of non-bouted MVPA, while these daily averages were 14.9 and 9.46 min using 5-min, and 8.1 and 4.5 min using 10-min bout MVPA. In adjusted analyses, men aged 80 years or more spent in average 45 min less LPA per day when compared to men 60-69 years and, among women, this difference was 65 min. Considering time in 5-min MVPA bouts, the youngest age group and those with a better self-perceived health accumulated more MVPA. Specifically among men, socioeconomic status was inversely associated with 5-min bout MVPA.	-
Bielemann et al. [26] 2018	To evaluate cross-sectional and longitudinal associations of consumption of dairy products and physical activity (PA) with bone mineral density (BMD).	Association with health variable	C2004, (6,8y), N = 2,636	Overall PA: ENMO vartiable provided in mg, MVPA in 10-min bouts	At 6 years, BMD was measured in 3444 children and 2636 children provided data on objectively measured PA by accelerometry. PA assessed by accelerometry was positively related to total-body and lumbar-spine BMD in boys and lumbar-spine BMD in girls. We did not find evidence for an interaction between PA and	-
Silva et al. [30] 2018	We assessed overall PA, moderate, vigorous, and moderate-to-vigorous physical activity (MVPA) objectively measured among pregnant women and their correlates in a population-based study.	Descriptive	C2015, pregnant women, N = 2,317 ActiGraph wGT3X-BT	Overall PA: ENMO vartiable provided in mg, MPA, VPA, MVPA non-bouted and in 1,5 and 10-min bouts	Overall PA was 27.6 mg. Pregnant women spent on average 14 min/day in MVPA and 0.4 min in vigorous PA. Time spent in MVPA and total PA were inversely associated with years in school and income, and were lower among women receiving advice to not exercise. MVPA was also inversely associated with age, lower among women living with a partner, and higher among non-white women. The study indicated low levels of PA among pregnant women. The identified correlates may provide a framework to better understand factors influencing PA during pregnancy and thus inform future interventions.	

Mielke et al. [40] 2018	Examine the associations between socioeconomic position (SEP) and self-reported and objective measures of sedentary behavior in adolescents, using a life-course approach.	Associations with health variable *	C1993,(18y), N = 3,589 GENEActiv	Sedentary behavior	In corss sectional analysis only maternal education was positively associated with screen time [OR = 1.51 (95%CI: 1.35; 1.68)]. In longitudinal analysis maternal education at birth, 11 y and 15y were associated to higher sedentary behaviour quintile. The same was found for income. EP at birth was positively associated with total screen time at 18 years, with most of this association mediated by SEP at other ages.	-
Bielemann et al. [29] 2019	To evaluate independent associations of moderate and vigorous physical activity (MPA, VPA) across adolescence with areal bone mineral density (aBMD).	Association with health variable	C1993, (18y), N = 3,542 GENEActiv	MPA, VPA in 5-min bouts	Lumbar spine and femoral neck a BMD were available for 3947 (49.9% of boys) and 3960 (49.6% of boys) individuals, respectively. For boys, MPA was associated to higher femoral neck in while VPA was associated to higher Lumbar spine and Femoral neck. For girls, only PA was associated to higher Lumbar spine and Femoral neck.	-
Silva et al. [32] 2019	We assessed the optimal number of days needed to obtain reliable estimates of overall PA and moderate-to-vigorous physical activity (MVPA) during the 2nd trimester in pregnancy using a raw triaxial wrist-worn accelerometer.	Descriptive	C2015, pregnant women, N = 2,082 ActiGraph wGT3X-BT	Overall PA: ENMO variable provided in mg, MVPA in 5-min bouts	Among 2,082 pregnant women who wore the accelerometer for seven complete days, overall and MVPA were lower on Sundays compared to other days of the week. Reliability of ≥ 0.80 to evaluate overall PA was reached with at least three monitoring days, whereas seven days were needed to estimate reliable measures of MVPA.	-
Müller et al. [33] 2020	This study aims to examine the associations between PA during pregnancy and occurrence of preterm birth.	Association with health variable	C2015, mothers, N = 1,963 ActiGraph wGT3X-BT	Overall PA: ENMO variable provided in mg	Multivariate analysis showed that objectively PA was not associated to higher risk of preterm birth. Self-reported PA in the tird trimester was associated to lower OR for preterm birth.	-
Ricardo et al. [43] 2019	Describe objectively measured physical activity (PA) and its correlates in one-year-old children.	Descriptive	C2015, (1y), N = 2,974 ActiGraph wGT3X-BT	MVPA in 10-min bouts, Sedentary behaviour	Infants able to walk independently spent on average 19 h per day below 50 mg of acceleration (including sleep time), and those who could not walk spent on average 21 h in this intensity category. Girls spent approximately 10 min more than boys below 50 mg daily in both walking status categories, and less activity than boys on higher intensity categories. Boys and infants whose mothers were more physically active during pregnancy presented	-

					more acceleration, regardless of walking status. Among infants who could walk by themselves, those with mothers with one to eight schooling years; adequate length-for-age (z-score); not attending daycare; and more physically active fathers also showed higher levels of acceleration.	
Silva et al. [32] 2019	Evaluate independent and combined cross-sectional associations of objectively-measured physical activity and sedentary time with body composition outcomes at 30 years, and prospective associations of changes in self-reported physical activity from 23 to 30 years with the same outcomes in participants from the 1982 Pelotas (Brazil) Birth Cohort.	Associations with health variable	C1982(23, 30y), N = 3,206 GENEActiv	Overall PA: ENMO variable provided in mg, LPA, MVPA in 5-min bouts	In cross-sectional analyses, higher objectively-measured MVPA was associated with lower body mass index (β = 0.017, 95%CI: -0.026; -0.009), waist circumference (β = -0.043, 95%CI: -0.061; -0.025), visceral abdominal fat (β = -0.006, 95%CI: -0.009; -0.003), and fat mass index (β = -0.015, 95%CI: -0.021; -0.009), independent of sedentary time. Sedentary time was independently associated only with higher fat mass index (β = 0.003, 95%CI: 0.001; 0.005). In longitudinal analyses, using self-reported measure, adiposity was lower among those who were consistently active or who became active. Adiposity was similar among the "became inactive" and "consistently inactive" subjects.	-
Bielemann et al. [27] 2020	Use of objectively measured physical activity (PA) in older adults to assess relationship between PA and risk of all-causes mortality is scarce. This study evaluated the associations of PA based on accelerometry and a questionnaire with the risk of mortality among older adults from a city in Southern Brazil.	Association with health variable	Cohort Como vai, (60y+), N = 971 GENEActiv	Overall PA: ENMO variable provided in mg, MPA, VPA, MVPA non-bouted, in 1,5 ans 10-min bout and ≥ percentile 90	From the 1451 older adults interviewed in 2014, 145 died (10%) after a follow-up of an average 2.6 years. Men and women in the highest tertile of overall PA had on average a 77% and 92% lower risk of mortality than their less active counterparts (95%CI: 0.06; 0.84 and 95% CI: 0.01; 0.65, respectively). The highest tertile of LPA was also related to a lower risk of mortality in individuals of both sexes (74% and 91% lower risk among men and women, respectively). MVPA statistically reduced the risk of mortality only among women (hazard ratio [HR] = 0.30 and HR = 0.07 in the second and third tertiles). Self-reported leisure-time PA was statistically associated with a lower risk of mortality only among men. Women in the highest tertiles of commuting PA showed a lower risk of mortality than	-

					those in the reference group.	
Ricardo et al. [44] 2020	To estimate the minimum number of accelerometer measurement days needed to estimate habitual physical activity (PA) among 6, 18 and 30- year-old participants, belonging to three population-based Brazilian birth cohorts.	Methodological	C1982,(30y), N = 452 C1993, (18y), N = 503 C2004, (6y), N = 103<br><br>GENEActiv	Overall PA: ENMO variable provided in mg, MVPA in 5-min bout, LPA	Differences between week and weekend days regarding LPA, MVPA and overall PA, were only observed among 30-year-olds. Higher levels of MVPA ($p = 0.006$) and overall PA ($p < 0.001$) were identified on weekdays. For overall PA, to achieve a reliability coefficient > 0.70, two and three days of measurement were needed in adults and children, respectively. For LPA, a reliability coefficient > 0.70 was achieved with five days in 6-year-old children, three days in 18-year-old young adults, and four days in 30-year-old adults. Considering MVPA, four days would be necessary to represent a week of measurement among all cohort groups.	-
Wendt et al. [46] 2020	Describe the sleep time window, total sleep time, and sleep percent by accelerometry in a population-based young adult cohort in Brazil.	Descriptive	C1993, (22y), N = 2,462 ActiGraph, wGT3X-BT	STW TST efficiency (sleep percentage)	The means of TST, STW, and SP for men were 5.9 h, 7.1 h, and 83.1%, respectively. For women, the means of TST, STW, and SP were 6.4 h, 7.6 h, and 84.6%, respectively. Women presented a higher means of all outcomes compared to men ($p < 0.001$). After adjusting for both sexes, white skin color and not working or studying were associated with higher TST. Individuals not working or studying presented higher means of STW and lower sleep SP. Women with children who were less than two years of age presented lower values of three evaluated outcomes. Regarding behavior and health condition variables, obesity was associated with lower STW only for men. Physical activity was associated with higher SP and risk drinking with lower TST and STW only for women.	-
Wendt et al. [47] 2022	To verify the effect of different intensities of PA practiced in different periods of the day on the subsequent sleep night in a population-based cohort of young adults.	Associations with health variable	C1993, (22y), N = 2,006 ActiGraph, wGT3X-BT	LPA and VPA (nonbouted), MPA in 5-min bout STW TST efficiency (sleep percentage)	The means of STW, TST, and SP were 443.6 min/day, 371.1 min/day, and 84%, respectively. Time spent in moderate PA and vigorous PA in the morning and afternoon was not associated with sleep variables. Among men, 10 min/day of morning LPA increased TST by 2.56 min/day. Among women, 10 min/day of morning LPA	In order to improve the quality of data, TST and STW of less than 2 h or more than 15 h, respectively, were excluded from the analysis.

					increased SP by 0.15 percentage points. Afternoon LPA also increased SP by 0.09 percentage points for women. Night PA seems to have an inverse effect on sleep variables for any intensity and both sexes.	
Wendt, A. et al. [48] 2020	To describe objectively measured physical activity (PA) in different periods of the day in young adults according to sex, socioeconomic position and during weekdays and weekends.	Descriptive	C1993, (22y), N = 2,766 ActiGraph, wGT3X-BT	LPA (nonbouted), MVPA in 5-min bout	LPA was higher among women, while bouted MVPA levels were higher among men. The median of PA was higher on weekdays compared to weekends for all intensities. The bouted MVPA medians in the morning and at night were zero minutes for all days and both sexes. The richest group presented a higher per-centage of individuals with zero minutes. PA may vary according to different periods of the day and intensity. The absence of PA practice was markedly influenced by sex and socioeconomic position.	Periods of the day were divided in morning, afternoon, and night.
Martins et al. [39] 2021	Describe patterns of losses of information regarding accelerometer data and to assess the use of multiple imputation to generate physical activity estimates for individu-als without accelerometry data.	Descriptive	C1993, (22y), N = 2,985 C2004, (11y), N = 3,348 ActiGraph, wGT3X-BT	Overall PA: ENMO variable provided in mg	Male individuals, participants with black skin color, and less schooled individuals presented higher averages of overall physical activity than their counterparts. Almost all imputed estimates were comparable to the complete cases, and the highest difference found was 0.7 mg for the first quintile of socioeconomic status of the 1993 birth cohort.	-
Mielke et al. [41], 2021	Investigate the cross-sectional and prospective associations between accelerometer-measured physical activity and cardiometabolic health in the transition to adulthood.	Associations with health variable	C1993,(18,22y), N = 2.280<br><br>ActiGraph, wGT3X-BT GENEActiv	Overall PA: ENMO variable provided in mg, MVPA in 1 and 10-min bout	Overall, inverse dose-response associations between MVPA and cardiometabolic health at age 18 and 22 yr were observed in cross-sectional analyses of data from men and women. Prospective analyses showed that, in general, MVPA declined, and cardiometabolic health worsened in this 4-yr period in both men and women. Cardiometabolic health at age 22 yr reflected both MVPA at age 18 yr (β = -0.007; 95%CI: -0.014; 0.000) and changes in MVPA from 18 to 22 yr (β = -0.030; 95%CI: -0.043; -0.016) in men, but only changes in MVPA in women (β, -0.035; 95%CI: -0.058; -0.011). In analyses of change over time, men who improved MVPA by 20 to	-

					30 min·d-1 showed significant improvements in cardiometabolic health over 4 yr. The magnitude of association was slightly stronger for MVPA in 10-min bouts than for MVPA accumulated in bouts of 1 min, especially in women.	
Bielemann et al. [28], 2022	This study evaluated prospective associations between self-reported and objectively measured physical activity (PA) and risk of falls among older adults.	Association with health variable	Cohort Como Vai, (60y), N = 971 GENEActiv	Overall PA: ENMO variable provided in mg, LPA, MVPA in 5-min bouts	Around 23% of the 1,161 participants followed-up in 2016-2017 experienced a fall in the last 12 months. Participants who did not spend any time in self-reported leisure-time PA at baseline had on average 34% higher risk of falls, and individuals in the lowest tertile for moderate to vigorous PA had on average 51% higher risk of falls compared to those in the highest tertile. Low levels of self-reported and objectively measured moderate to vigorous PA were related to higher risk of falling among Brazilian older adults.	-
Leão et al. [38], 2022	Investigate longitudinal associations between physical activity and early childhood neurodevelopment.	Artigo pago doi: 10.1123/jpah.2021-0587	C2015, (1,2,4y), N = 1,673 ActiGraph, wGT3X-BT	Overall PA: ENMO variable provided in mg	Of the 3 physica activity trajectories observed, children in the medium (β = 1.17; 95%CI: 0.25; 2.10) and high (β = 2.22; 95%CI: 0.61; 3.82) trajectories showed higher neurodevelopment scores than children in the lower activity trajectory. Cumulative analyses showed that children in the highest tertile of physical activity in all follow-ups presented a mean neurodevelopment score 4.57 (95%CI: 2.63; 6.51) higher than children in the lowest tertile in all follow-ups. All analyses showed a dose-response characteristic of association, with higher physical activity indicating higher neurodevelopment scores.	-

* Socioeconomic position was considered a health variable in this study.

LPA: light physical activity; MPA: moderate physical activity; MVPA: moderate to vigorous physical activity; VPA: vigorous physical activity.

Supplementary Material 2: Code used in analyses

```
graphics.off() #Ensure that there are no graphic devices going on
rm(list=ls())  #Removing all variables/objects from the working environment
library(GGIR)

##############
#INPUT NEEDED#
##############

datadir = "example" # directory where accelerometer files are / list of accelerometer files incl. path
studyname = ""#"mystudy" #name of sutdy, only needed if datadir is a list of filenames
outputdir = "example" #name directory where output needs to be stored
overwrite=FALSE

f0 = 1 #file to start with
f1 = length(list.files(datadir)) #file to end with if used in serial analyses (modify accordingly, if infinite then it will process until last file)

####################################
#NO INPUT NEEDED FROM HERE ONWARDS#
####################################

#Function (named my.g.shell.GGIR) that will be used to run modes 1-5
my.g.shell.GGIR <- function(mode, f0, f1) {
  desiredtz ="America/Sao_Paulo"
  g.shell.GGIR(#-------------------------------
        # General parameters
        #-------------------------------
        mode=mode,
        datadir=datadir,
        outputdir=outputdir,
        studyname=studyname,
        f0=f0,
        f1=f1,
        overwrite = FALSE,
        do.imp=TRUE,
        idloc=1,
        print.filename=TRUE,
        storefolderstructure = FALSE,
        #-------------------------------
        # Part 1 parameters:
        #-------------------------------
        windowsizes = c(5,900,3600),
```

```
do.cal=TRUE,
do.enmo = TRUE,
do.anglez=TRUE,
chunksize=1,
printsummary=TRUE,
#-------------------------------
# Part 2 parameters:
#-------------------------------
strategy = 1,
ndayswindow=7,
hrs.del.start = 1,
hrs.del.end = 1,
maxdur = 9,
includedaycrit = 16,
L5M5window = c(0,24),
M5L5res = 10,
winhr = c(5,10),
qlevels = c(c(1380/1440),c(1410/1440)),
qwindow=c(0,24),
ilevels = c(seq(0,400,by=50),8000),
mvpathreshold =c(100,200,300,400,500,600,700,800),
#-------------------------------
# Part 3 parameters:
#-------------------------------
timethreshold= c(5),
anglethreshold=c(3),
ignorenonwear = TRUE,
#-------------------------------
# Part 4 parameters:
#-------------------------------
#Labels detected sustained inactivity periods by g.part3 as either nocturnal sleep or daytime sustained inactivity
#Description
#Loads output from g.part3 as stored in milestone data and sleep log information (if available) and
#then uses these information sources to define nocturnal sleep and daytime sustained inactivity.

excludefirstlast = FALSE,
includenightcrit = 16,
def.noc.sleep = 1,
loglocation= c(),
outliers.only = FALSE,
relyonsleeplog = FALSE,
sleeplogidnum = TRUE,
colid=1,
coln1=2,
do.visual = TRUE,
```

```
	#relyonsleeplog: If TRUE then sleep onset and waking time are defined based on timestamps derived from sleep log
	#if FALSE (default) the sleep log is only used to guide the accelerometer-based detection.
	#If participants were instructed NOT to wear the accelerometer during waking hours then set to TRUE, in all other
scenarios set to FALSE (FALSE).

	sleeplogidnum = TRUE,
	colid=1, #Column number in the sleep log spreadsheet in which the participant ID code is stored (default = 1)
	coln1=2, #Column number in the sleep log spreadsheet where the onset of the first night starts

	do.visual = TRUE,
	#nnights=5, #Number of nights for which sleep log information should be available.
	#It assumes that this is constant within a study. If sleep log information is missing for certain nights then leave these blank
	sleeplogidnum=TRUE,
	#-------------------------------
	# Part 5 parameters:
	#-------------------------------
	# Key functions: Merging physical activity with sleep analyses
	threshold.lig = c(30,40,50),
	threshold.mod = c(100,200,300,400,500,600,700,800),
	threshold.vig = c(400,500,600,700,800),
	boutcriter = 0.8,
	boutcriter.in = 0.9,
	boutcriter.lig = 0.8,
	boutcriter.mvpa = 0.8,
	boutdur.in = c(10,20,30),
	boutdur.lig = c(1,5,10),
	boutdur.mvpa = c(1,5,10),
	timewindow = c("WW"),
	#-----------------------------------
	# Report generation
	#-------------------------------
	do.report=c(2,4,5))
}

#############
#RUN PHASE 1#
#############

#To run in 60 cores in parallel
library(doParallel)
registerDoParallel(60)
my.g.shell.GGIR(mode=1, f0=f0, f1=f1)

###############
#RUN PHASE 2 - 5#
```

```
###############
my.g.shell.GGIR(mode=2, f0=f0, f1=f1)
my.g.shell.GGIR(mode=3, f0=f0, f1=f1)
my.g.shell.GGIR(mode=4, f0=f0, f1=f1)
my.g.shell.GGIR(mode=5, f0=f0, f1=f1)
```

Supplementary Material 3: Examples of a usual data (A) and abnormal data (B).

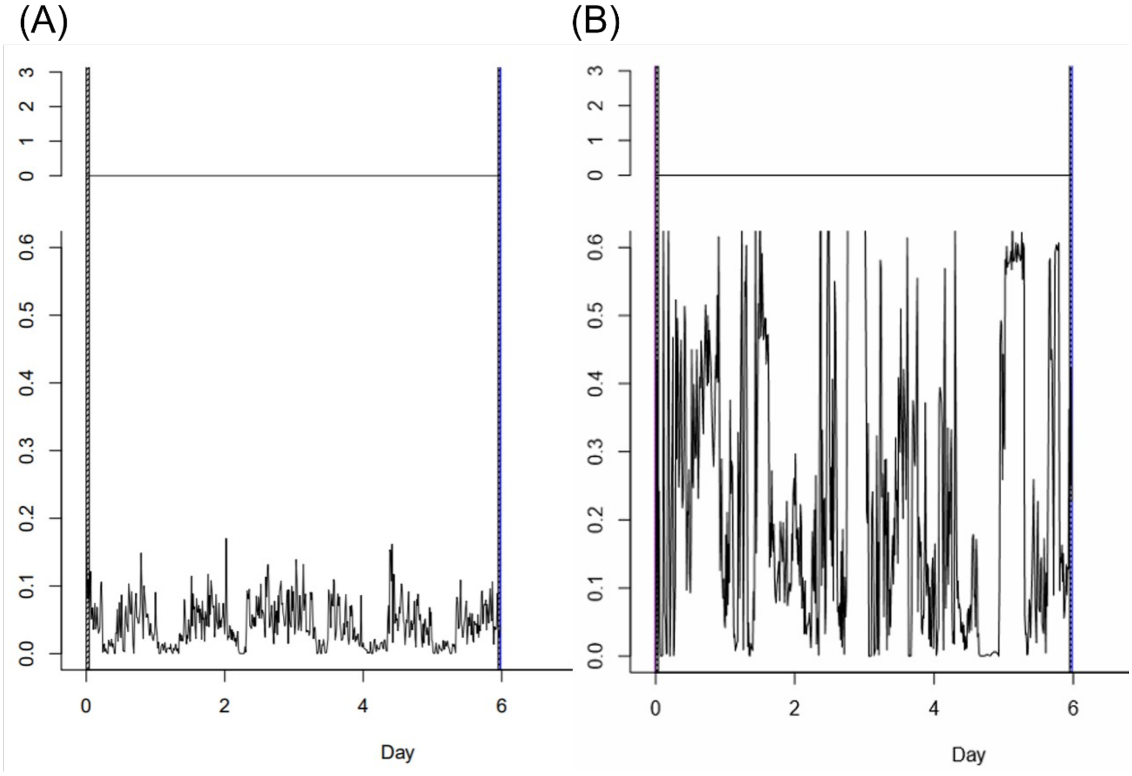

Supplementary Material 4: Definition of main variables sent to dataset manager of each cohort.

VARIABLE	DEFINITION
tst	Total sleep time – Total of minutes classified as sleep between sleep onset and slep end
stw	Sleep time window – Difference (in minutes) between sleep onset and sleep end
sleep_percentage	Ratio between tst and stw. Expessed in % give an idea of 'quality of sleep'. This variable is similar to sleep efficiency, but GGIR not detects sleep latency without a sleep log. For this reason this varable is called sleep_percentage.
overall_pa	Daily acceleration avergae (based on ENMO metric and expressed in mg)
i_stability	Interdaily stability- indicates the sincronization of rest-activity rhythm
i_variability	Intradaily variability- indicates fragmentation of rest-activity rhythm
mvpa	Daily average of minutes spent in moderate to vigorous activities (>100mg)
mvpab1	Daily average of minutes spent in moderate to vigorous activities (>100mg) considering bouts of 1 minute
mvpab5	Daily average of minutes spent in moderate to vigorous activities (>100mg) considering bouts of 5 minute
mvpab10	Daily average of minutes spent in moderate to vigorous activities (>100mg) considering bouts of 10 minute
inatb30	Daily minutes spent in susteined inactivity in bouts of 30 minutes (inactive 90% of these bouts)
Inatb20	Daily minutes spent in susteined inactivity in bouts of 20 minutes (inactive 90% of these bouts)
Inatb10	Daily minutes spent in susteined inactivity in bouts of 10 minutes (inactive 90% of these bouts)
inat	Inactivity – Daily minutes average spent in activites with <50mg (excluding sleep). This is na usual definition of sedentary behaviour but device used in cohort have no inclinometer. Due this reason this variable is called inactivity.

Supplementary Material 5: Detailed flow chart for each follow-up in each cohort

a) C1982 – 30 years

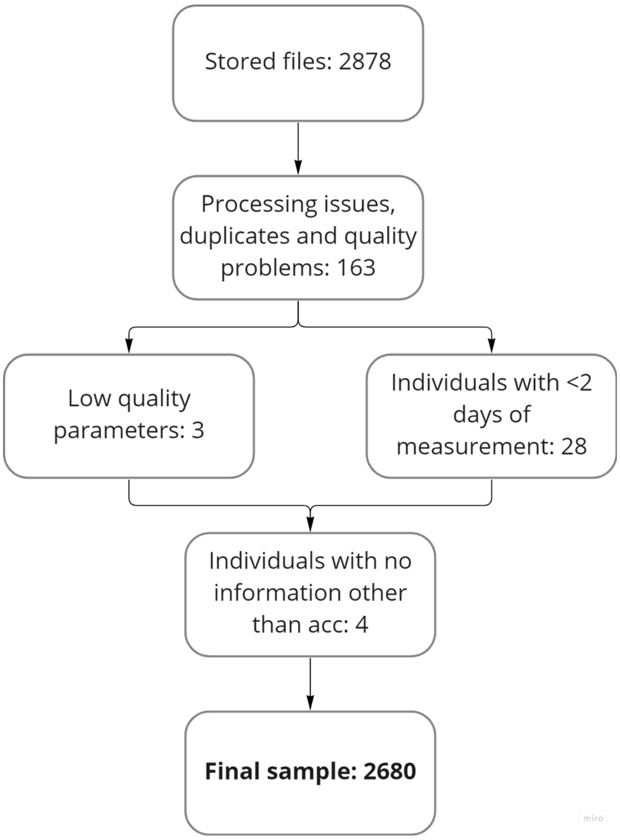

b) C1993 – 18 years

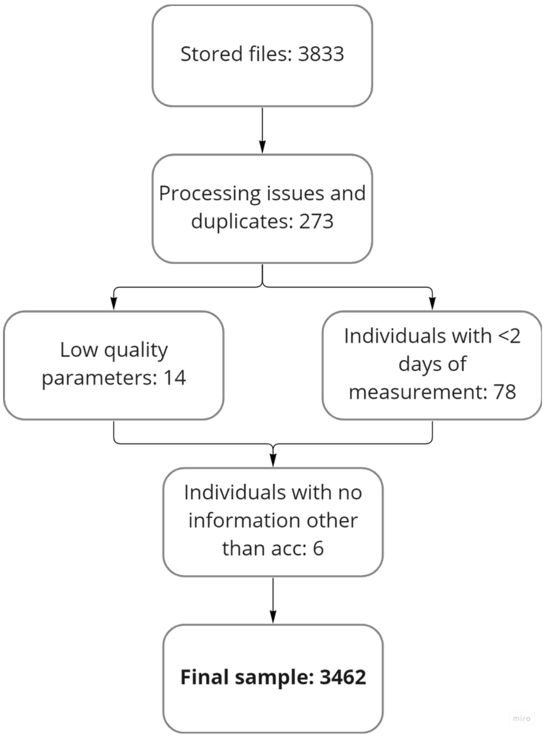

c) C1993 – 22 years

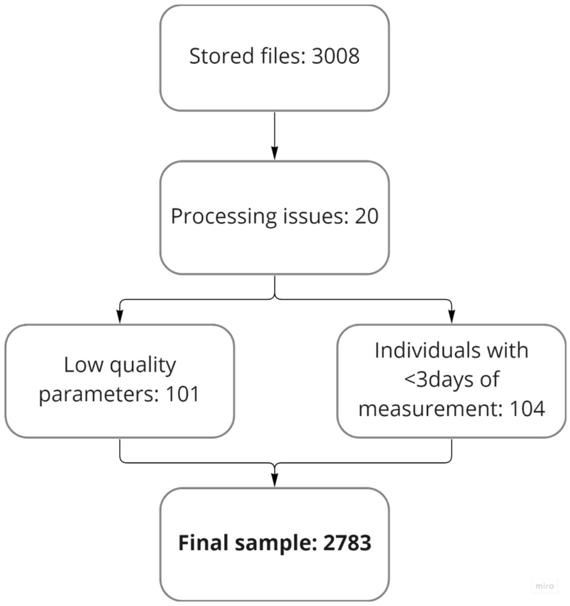

d) C2004 – 7 years

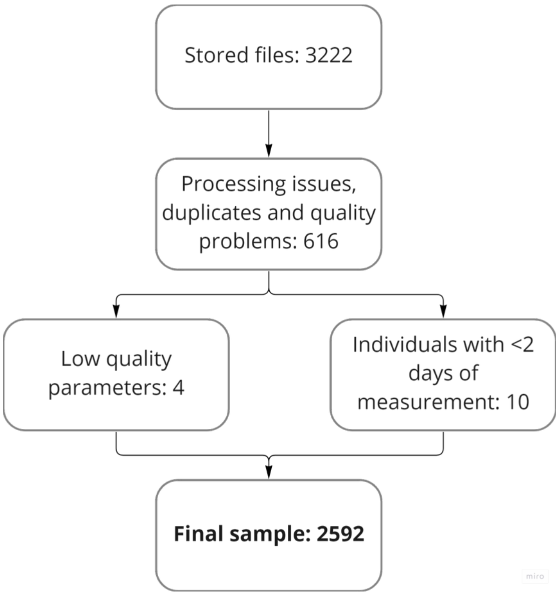

e) C2004 – 11 years

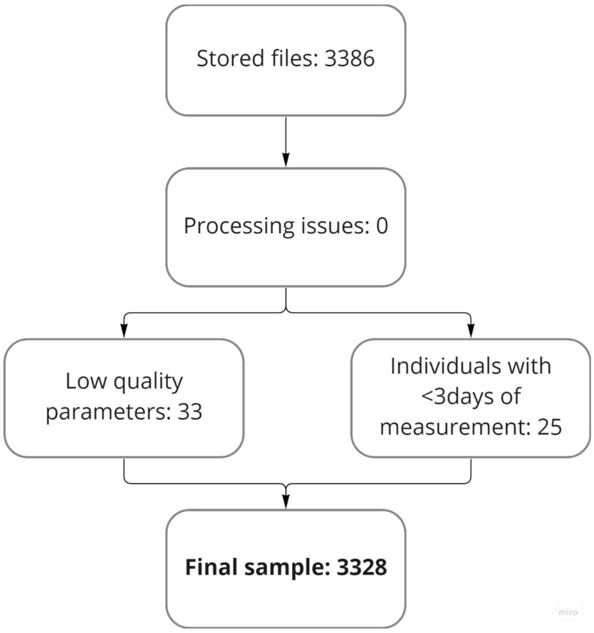

f) C2004 – 15 years

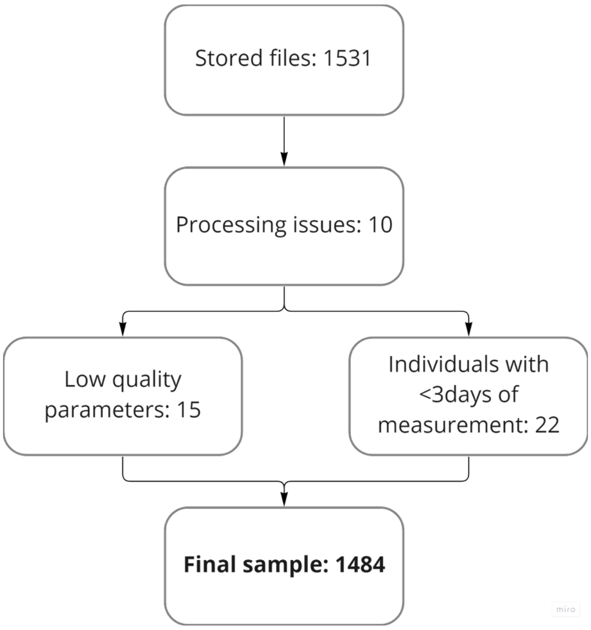

g) C2015 – mother (gestation)

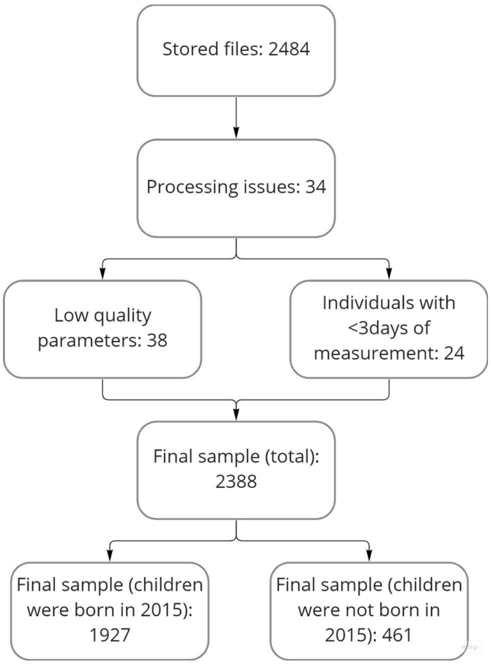

h) C2015 – mother (2 years of child)

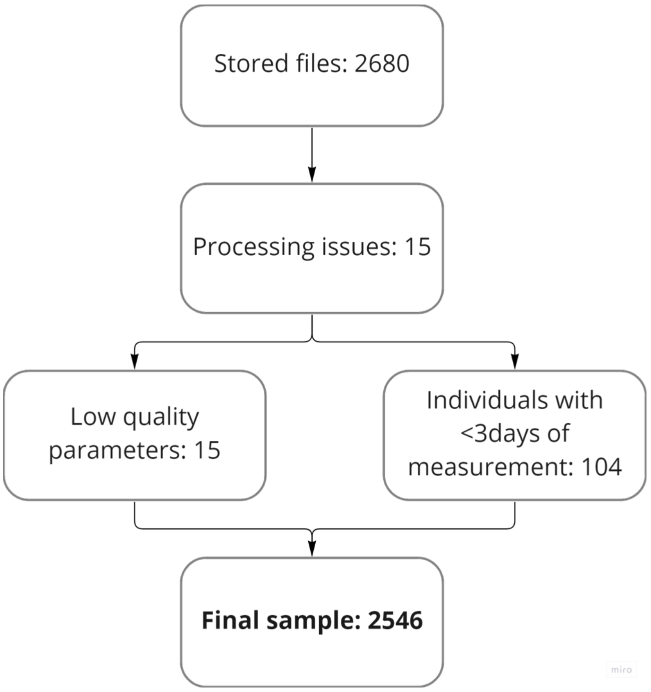

I) C2015 – father (1 year of child)

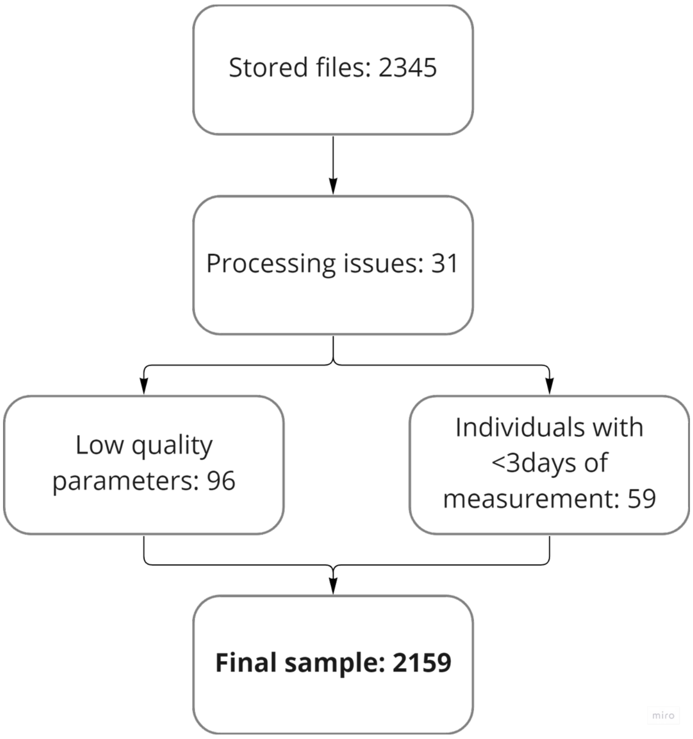

j) C2015 – 1 year

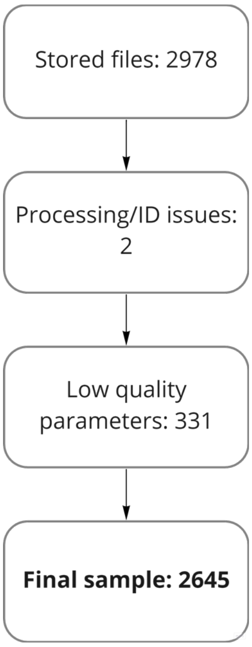

k) C2015 – 2 years

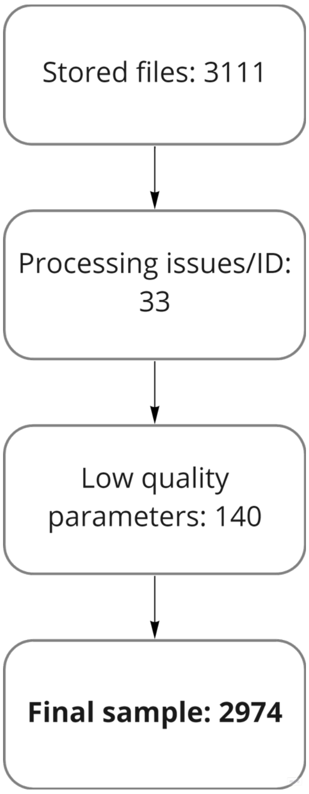

l) C2015 – 4 years

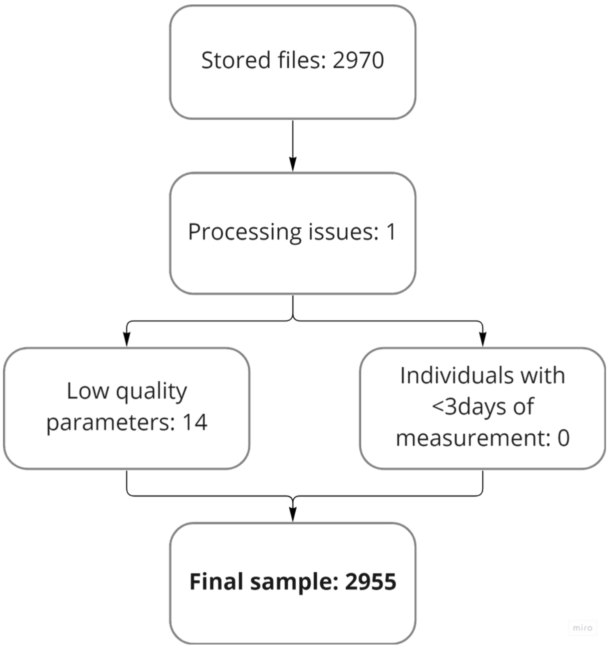

m) *"Como vai?"* study

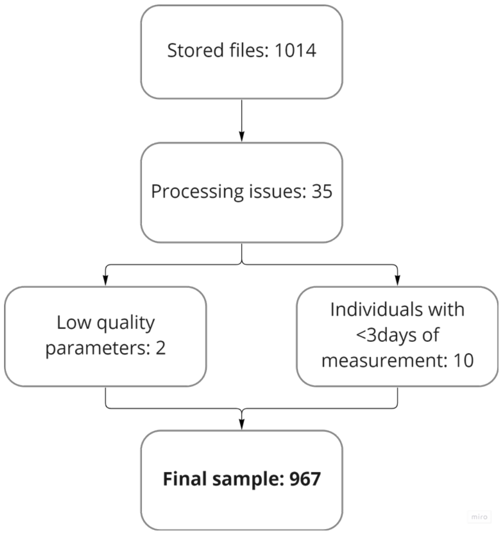

Supplement: Supplementary file 1 [file 1678-4464-csp-41-03-EN011724-s.pdf]
